# Surgery of the War

**Published:** 1855-04

**Authors:** 


*Surgery of the War.*—Napoleon, at Eylau, taking a diamond star placed it on that of a young medical officer. In a deadly charge the day before, we are told, thousands were wounded; at last the serried lines of the French gave away, and retreated by a series of manœuvres, in one of which, amongst dead and dying, a surgeon was seen, suddenly called to a general terribly wounded. A large artery was open; cold and harassed, the surgeon knelt by his patient; shouts were raised on all sides for him to save himself. The battalions of the enemy literally rode over him; the bullets of the opposing army whistled in hundreds by his ears; still he pressed on the artery, and ultimately saved the life of the young officer. A bitter cold night followed a more frightful day. The surgeon crunched the snow in his hand, and applied it to the wound. Something little short of a miracle had occurred, but the surgeon never deserted his post; and on Napoleon seeing him next day the diamond cross was placed on his breast. A few months ago a tourist in France saw this cross on the coffin of an old village surgeon, and heard the story.

All honor to our military surgeons! The French can boast of this old Dr. Becourt, and Pontier, the Larrey of their present hospitals at Sebastopol; we can feel a pride, also, in the names of Mackenzie, Williams and Thomson. It is too true we have no decorations or honors for our medical men, but their names and deeds live enshrined in the hearts of all who esteem a manly appreciation of duty, truth, and of Christian bravery; for who is it in the dread field of death and battle, though wearied and worn in spirit himself, that can carry such hope and animation to the mind of the wounded soldier, if it be not the surgeon? Who is it that is expected to be awake at all hours, while others sleep—who is it, while night falls drearily on tent, camp and field, as Homer delights to tell us, still works and watches, and inspires hope and confidence? If accident had not led this tourist into one of the little villages of France lately, we should not have had this anecdote (to be found in one of the French daily newspapers). Far from the noise of the outer world, neglected and forgotten, this poor surgeon died; and yet this rare devotion, this true heroism, this life of mental thought and overwhelming work, do not attract the crowd. The great general or diplomatist, the Minister of the War-office, &c., have all or each a peerage or pension; but the surgeon is—we will not say forgotten; for here, at least, in the medical journal, we must take note of such generous deeds, such noble devotedness, while in their writings will Larrey, Guthrie and Ambrose Perie be never forgotten. Parliament has now assembled, and we trust a better feeling will be apparent towards our navy and army medical officers. Five hundred wounded more are added to the hospital at Scutari, rescued from the ambulances and tents all blown down in the storm of last month. We learn, however, that an hospital ship, the New York, has been despatched with seventy-five ambulance carriages, vegetables to prevent scurvy, and various medical necessaries.

The charge, the rout, the flying squadrons, the glittering colors, and other "pomp and circumstances" of war, are no doubt very grand. The martial music of the marching army; the kings and generals and their staff; the surgeon meanwhile, perhaps, on foot unnoticed. But a day comes when the surgeon takes the place of this popular display; that is the day after the battle. It is for him now a peculiar glory to be calm when all others are excited.

There is more of interest and beauty then for him in the resources of anatomy, and in the wise arrangements of nature in healing wounds. He may have galloped with cavalry, as Dr. Wilson, lately into the thickest of the enemy; but in his hospital no emotion must influence his hand in operating. Russian, French and English patients are all alike; he must know what to say to the poorest soldier, as well as to the greatest general. We know few men, in fact, that require to be so much masters of general knowledge. We have said that he will find much of beauty in his art and in the arrangements of nature in healing wounds; he will find also much to influence him in the parting words of the dying—secrets confided to his keeping, and last words to be conveyed to the loved ones far away. The surgeon must decline no office. The young military surgeon must be a thorough man of thought and feeling; the joyous companions of former times, officers he has joked with perhaps in former regiments, are dying, and require one manly, Christian word of a religious kind. He must control his emotions as a surgeon; he must feel deeply as a friend. One tear betrays the man, but less than a tear takes away the *prestige* of the soldier. There is a country and a cause to be fought for, above every other consideration. At the plague of Jaffa, it is told, the soldiers all refused to fight, fearing the contagion of this disease. In vain the generals harangued. The skeleton figures of those soldiers who had caught the plague terrified the entire army, till Desgenetts, a surgeon, offered to sleep with those stricken with the plague, and inoculated himself with it, even under the bullets of the enemy! Taken prisoner afterwards, this noble act was even respected by the enemy, and his life saved. Here is a man, then, all but sacrificing himself to a sense of duty. Yes, the military surgeon has much of moral grandeur in his quieter conquests, superior even to that of the battle field; kneeling by the straw bed of the ambulance; bent double to reach the ground, he has myriad thoughts to engross his mind, a crowd of contending emotions to regulate; he has to inspire hope, and yet feel as one without hope; his unbroken moral courage must do many things for the poor soldier, struck down and dying, which the world never hears about; he is the great repository of the farewell secrets of the wounded, of a favorite ring, or note, or seal, great things or small; he is expected to manage everything. Like a sentry, the surgeon watches at all hours, and when the morrow arrives he is still to keep watch and ward in the trenches, or on the grim battle-field itself; he has to improvise all sorts of resources; he is at once carpenter and nurse, secretary and soldier, physician and friend.

On the 21st of September the surgeon of the Vulcan, quite unexpectedly, received on board his small ship 500 wounded from the battle of Alma. Dressings, lint, calico, &c., were wanted; but the sailors and officers gave all their sheets, shirts, &c., to the wounded, so great was the moral influence of the surgeon over them. The surgeon of the Agamemnon came on board, but it was midnight before they were all dressed. Another surgeon had to operate several times without chloroform, amputate thighs, his orderly or servant alone assisting him. The Minie rifle bullet splits and smashes the bone, while the old bullet bored holes in it. A dozen instances are given of surgeons left to shift as best they can, without ambulances, without wine, without lint or bandages; the moral courage of such men beats down all difficulties; the character of the true surgeon wins all hearts, triumphs over every disaster.—*London Lancet.*

